# SJA is now on PubMed

**DOI:** 10.4103/1658-354X.65116

**Published:** 2010

**Authors:** Abdulhamid Al-Saeed

**Affiliations:** *Editor-in-Chief, Saudi Journal of Anaesthesia E-mail: editor@saudija.org*

It gives me great pleasure to announce that the Saudi Journal of Anaesthesia has now been indexed on PubMed Central (PMC) and PubMed list. We received this news on June 9, 2010. Indeed, it is an excellent achievement and it represents the hard and continuing work of the editorial board. Let me give you a brief on the process of publication, once an article is received online. Following online submission, the Editor in Chief (EIC) assigns an editor for managing the manuscript. This editor checks the outlines of the manuscript and if the author has not followed the instructions given in the journal, the editor transfers the article to technical modification square and the article is then sent back to the author. Upon receiving the corrected version, the editor checks it for plagiarism. If the manuscript passes the plagiarism test, then reviewing process will start. It took us considerable time to build our reviewers‘ database and we feel proud of having excellent reviewers representing different anaesthesia subspecialties. The article is sent to three external reviewers and we ask them to send their report online within 2 weeks from the day they are contacted. Honestly, we are proud of having such a standard of professional reviewers and let me take this opportunity to thank all of them for their dedicated time in reviewing the manuscripts. Upon receiving the three reviewers‘ reports, we send them to the author for revision. The manuscript moves between external re-review and revision squares, till a provisionally accepted version is obtained. After this, the article is sent to the pre-accepted language check square which is represented by our publishing office “Medknow Publications". Once language is checked, the article is sent to the EIC for a final decision and scheduling for a certain issue/year of SJA. I wish to take this opportunity to extend my thanks and appreciation to “Medknow Publications” staff office and their crew for their excellent and professional work to maintain the high standard of the SJA. Once an issue is completed, a proof will be prepared by the publishing office and sent to EIC for final approval. With the reviewing process described above, we ensure that the article is peer reviewed and published within a short period of time. Also, our reviewing process and plagiarism feature has enabled us to discover a plagiarized article and after consultation with our consulting editors we followed the slow judge procedure. This procedure started by informing the author of his unethical attitude. Since we received no answer for our queries, we raised the matter to his department chief, and further, to his college dean office, for appropriate action to be taken. In the meantime, we have sent a notice to Editors-in-Chief of most anaesthesia journals with the documents of the plagiarized article as a caution notice for any possible future submissions from his side.

Our short term plan in the future is to continue the hard work to maintain the high standard of the journal. We will continue producing three issues per year till the end of 2011, and starting from 2012, we will produce one more issue to achieve a total four issues per year. Our long-term plan is to be in the Institute for Scientific Information (ISI) list of journals, which we believe could be achieved.

I am proud of our editorial board members and I wish to thank them all for their time and efforts which brought the SJA to the international arena. My deep thanks and appreciation go to Professor Abdelazeem Eldawlatly for his continuing work and endless efforts to bring our dream to reality. Also, my deep thanks and appreciation are extended to the secretarial office of SJA and Saudi Anaesthesia Association for their tremendous efforts to achieve the association goals. Last, but not the least, I would like to thank all authors who published their work in SJA knowing that it was not indexed. The confidence that they showed in SJA and the support that they gave will remain unforgettable. Also, I wish to encourage all of you to submit your articles to SJA, without which we will be unable to upgrade our journal.

